# TMC1 may be a common gene for nonsyndromic hereditary hearing loss in Indian population

**DOI:** 10.1186/1755-8166-7-S1-P70

**Published:** 2014-01-21

**Authors:** Pawan Kumar Singh, Shipra Sharma, Manju Ghosh, Shivaram S Shastri, Neerja Gupta, Madhumita Roy Chowdhury, Madhulika Kabra

**Affiliations:** 1 Division of Genetics, Department of Pediatrics, All India Institute of Medical Sciences, New Delhi, India

## Background

Hearing impairment is very heterogeneous and most common sensory disorder. The prevalence of prelingual hearing loss is 1:500, with both environmental and genetic factors being equally responsible. To date, more than 128 loci and 74 genes responsible for nonsyndromic hereditary hearing loss have been identified, of which GJB2 gene is the most common across populations. Transmembrane channel like 1 or transmembrane cochlear-expressed gene 1 (TMC1) at DFNB7/11 locus at 9q13–q21 is another gene that is responsible for prelingual, severe to profound hearing impairment. It contains 24 exons and encodes 760 amino acids long 87.8 kDa multipass transmembrane protein, which is required in maintaining electrochemical homeostasis, structure and function of neurosensory hair cells in the inner ear. More than 29 different mutations have been reported in 48 families.

## Materials and methods

DNA was extracted from 47 multiplex families. Sanger sequencing identified connexin 26 mutations in six families and in the rest of the 41 families, homozygosity mapping was done using 35 fluorescent markers for eight common loci. DNA sequencing of TMC1 gene was done in all individuals of two families, in which linkage to DFNB7/11 locus was seen.

## Results and conclusion

Linkage to DFNB7/11 by four markers was found in two families. DNA sequencing of TMC1 gene in this locus identified a reported homozygous mutation, c.100C>T (p.R34X) in one family. In the other family, a novel homozygous change, c.1283C>A (p.Ala428Asp) was found in all the affected children. The mutation segregated with the hearing loss in both the families. Online protein prediction software SIFT and PolyPhen 2, predicted this novel change as damaging and probably damaging respectively. As TMC1 gene mutations have also been reported earlier in Indian and Pakistani families, it appears that TMC1 may be a common gene after GJB2 in the Indian subcontinent. c.100C>T is a common mutation in this gene. TMC1 gene should be considered in routine diagnosis if GJB2 is negative.

